# Hoxa9 Transduction Induces Hematopoietic Stem and Progenitor Cell Activity through Direct Down-Regulation of Geminin Protein

**DOI:** 10.1371/journal.pone.0053161

**Published:** 2013-01-11

**Authors:** Yoshinori Ohno, Shin'ichiro Yasunaga, Salima Janmohamed, Motoaki Ohtsubo, Keita Saeki, Toshiaki Kurogi, Keichiro Mihara, Norman N. Iscove, Yoshihiro Takihara

**Affiliations:** 1 Department of Stem Cell Biology, Research Institute for Radiation Biology and Medicine, Hiroshima University, Hiroshima, Japan; 2 Ontario Cancer Institute, McEwen Centre for Regenerative Medicine, Departments of Medical Biophysics and Immunology, University of Toronto, Toronto, Ontario, Canada; 3 Department of Food and Fermentation Science, Faculty of Food Science and Nutrition, Beppu University, Beppu, Oita, Japan; 4 Department of Hematology and Oncology, Research Institute for Radiation Biology and Medicine, Hiroshima University, Hiroshima, Japan; Emory University, United States of America

## Abstract

Hoxb4, a 3′-located Hox gene, enhances hematopoietic stem cell (HSC) activity, while a subset of 5′-located Hox genes is involved in hematopoiesis and leukemogenesis, and some of them are common translocation partners for Nucleoporin 98 (Nup98) in patients with leukemia. Although these Hox gene derivatives are believed to act as transcription regulators, the molecular involvement of the Hox gene derivatives in hematopoiesis and leukemogenesis remains largely elusive. Since we previously showed that Hoxb4 forms a complex with a Roc1-Ddb1-Cul4a ubiquitin ligase core component and functions as an E3 ubiquitin ligase activator for Geminin, we here examined the E3 ubiquitin ligase activities of the 5′-located Hox genes, Hoxa9 and Hoxc13, and Nup98-Hoxa9. Hoxa9 formed a similar complex with the Roc1-Ddb1-Cul4a component to induce ubiquitination of Geminin, but the others did not. Retroviral transduction-mediated overexpression or siRNA-mediated knock-down of Hoxa9 respectively down-regulated or up-regulated Geminin in hematopoietic cells. And Hoxa9 transduction-induced repopulating and clonogenic activities were suppressed by Geminin supertransduction. These findings suggest that Hoxa9 and Hoxb4 differ from Hoxc13 and Nup98-Hoxa9 in their molecular role in hematopoiesis, and that Hoxa9 induces the activity of HSCs and hematopoietic progenitors at least in part through direct down-regulation of Geminin.

## Introduction

Hox genes are clustered in four separate chromosomes (Hoxa-d), and are classified into 13 paralogous family members [1]. The Hox gene products determine the segment specificity during animal development and are also known to be involved in hematopoiesis and leukemogenesis, which are believed to be mediated by their transcription-regulatory activity [2],[3]. Hoxb4 and Hoxa9, the 3′- and 5′-located Hox gene respectively, enhance hematopoietic stem cell (HSC) activity [4],[5]. High levels of Hoxa9 expression are consistently seen in leukemic cells with the rearranged mixed lineage leukemia (Mll) gene [6], because Hoxa9 is a direct target gene for Mll fusion proteins [7]. Enhanced expression of Hoxa9 was shown to be essential for proliferative advantage and survival in leukemic cells [8]. Moreover, expression levels of Hoxa9 correlate well with poor prognosis for patients with acute myeloid leukemia [9]. Elevated Hoxa9 levels were also detected in the majority of patients with chronic myelogenous leukemia in the blast crisis phase [10]. In mice, Hoxa9 transduction was shown to enhance HSC activity and to suppress lymphoid differentiation [5]. Hoxa9 transduction was found to give rise to leukemic transformation, which, however, occurred 3 to 10 months after the transplantation, suggesting requirement of an additional genetic or epigenetic alteration for the leukemic transformation [5]. Some of the 5′-located Hox genes (*e.g.*, Hoxa9, Hoxc13 and Hoxd13) are partner genes for the chromosome translocations with Nup98 and are involved in leukemogenesis [11]. Transduction of either Nup98-Hoxa9 or Nup98-Hoxd13 caused myelodysplastic syndrome which progressed to acute leukemia after long latency periods [12],[13]. Similar leukemic transformation was observed in transgenic mice carrying Nup98-Hoxd13 [14] and Nup98-Hoxa9 [15]. Although many attempts have been made to identify the down-stream target genes for Hox gene derivatives [16],[17],[18],[19], the molecular role of these derivatives in hematopoiesis and leukemogenesis remains largely unknown.

We previously proposed that Polycomb-group (PcG) complex 1 (also designated as Polycomb repressive complex 1) sustains HSC activity through the E3 ubiquitin ligase activity for Geminin [20],[21],[22]. Interestingly, we found that retrovirus-mediated Hoxb4 transduction effectively restored the HSC defect caused by accumulated Geminin in mice deficient in Rae28 (also designated as Phc1), a member of PcG complex 1 [23]. We further provided evidence that Hoxb4 formed the RDCOXB4 complex with Roc1(also known as Rbx1)-Ddb1-Cul4a, a core component of E3 ubiquitin ligase, and that the RDCOXB4 complex acted as an E3 ubiquitin ligase for Geminin. Transduced Hoxb4 is presumed to down-regulate the accumulated Geminin via the ubiquitin-proteasome system (UPS) and to restore the impaired HSC activity in Rae28-deficient mice [23]. We therefore proposed that PcG complex 1 and Hoxb4 regulate the activity of HSCs through direct regulation of the Geminin protein [21],[23]. Geminin forms a Cdt1-Geminin complex to tune up the activity of Cdt1, which initiates DNA replication licensing by loading the MCM2-7 helicase onto chromatin [24]. Geminin acts as an inhibitor for Cdt1 and the ratio of Geminin versus Cdt1 controls “turning on and off” of DNA replication licensing [25]. DNA replication licensing occurs at late M and G_1_ phases and may also be involved in G_0_-to-G_1_ transition [24]. Geminin prevents rereplication from S phase to early M phase, which ensures one-round DNA replication in a single cell cycle [24],[26]. Geminin also acts as an inhibitor for Brahma and Brg1, a catalytic subunit of chromatin remodeling complexes, to maintain an undifferentiated state [27],[28] and as a transcription repressor or co-repressor [29],[30],[31]. Geminin deficiency abrogated early mammalian development at the blastocyst stage [32]. Conditional knockout of Geminin in hematopoietic cells perturbed the pattern of blood cell production [33]. Geminin is unstable protein which is present from S phase to late M phase and is degraded at the metaphase/anaphase transition by UPS in conjunction with the Anaphase Promoting Complex/Cyclosome (APC/C) in somatic cells [34]. Geminin is, on the other hand, known to be required for maintaining pluripotency at G_1_ phase in mouse embryonic stem and carcinoma cells [35]. Geminin expression is high in HSCs and is down-regulated in hematopoietic progenitors [21],[36]. Retrovirus-mediated Geminin transduction impaired clonogenic and replating activities of hematopoietic progenitors, while siRNA-mediated knock-down of Geminin promoted the progenitor activities [21],[23]. The dynamic expression of Geminin may thus at least provide HSCs with quiescence and hematopoietic progenitors with higher proliferation potential [21].

In the current study, we first conducted an *in vitro* examination by using recombinant molecules to determine whether the 5′-located Hox genes, Hoxa9 and Hoxc13, or Nup98-Hoxa9 generated the E3 ubiquitin ligase activity for Geminin, which is similar to the action by Hoxb4. We next examined the effect of the Hox derivatives on the Geminin protein and the cell cycle in a cell line derived from human kidney cells, HEK-293 cells and bone marrow cells (BM). We also assessed the involvement of down-regulated Geminin in hematopoietic stem and progenitor activities induced by Hoxa9 transduction. Based on our findings, we here argue for a novel molecular role of Hoxa9 in hematopoiesis and also discuss the possible involvement in leukemogenesis.

## Materials and Methods

### Real-time PCR

Total cellular RNA extracted from cells with the Mini RNA Isolation Kit (ZYMO Research, Orange, CA) was reverse transcribed by using TaqMan Reverse Transcription Reagents (Life Technologies, Carlsbad, CA). The resultant product was analyzed by means of real-time quantitative PCR analysis using TaqMan Gene Expression Assays and an Applied Biosystems 7500 Real-time PCR system (Life Technologies) and the specific transcripts were normalized to those of ß-actin.

### Transfection experiments

cDNAs were subcloned down-stream of the CMV promoter in pcDNA expression vector (Life Technologies). The plasmids were transfected with the calcium phosphate co-precipitation method into HEK-293 cells, which had been grown in Dulbecco's modified Eagle's medium (DMEM)(Life Technologies) supplemented with 10% FBS (ThermoFisher Scientific, Waltham, MA). The resultant transfectants were then further analyzed.

### siRNA experiments

HEK-293 cells were transfected with the following four double-stranded (ds) RNAs (ThermoFisher Scientific) at 40 nM with the aid of Lipofectamine RNAiMAX (Life Technologies) to knock-down Cul4a: RNAs—GCACAGAUCCUUCCGUUUA, GCAUGUGGAUUCAAAGUUA, GCGAGUACAUCAAGACUUU, and GAACAGCGAUCGUAAUCAA. siPerfect Negative Control (Sigma-Aldrich, St. Louis, MI) was transfected at the same concentration as non-target negative control [23]. On the other hand, mouse BM were cultured in DMEM supplemented with 15% FBS, 100 ng/ml mouse SCF, 100 ng/ml human TPO and 100 ng/ml mouse Flt3 ligand (R&D systems, Minneapolis, MN) for 24 hrs. Cells (5×10^5^) were harvested and resuspended in 1 ml Accell siRNA delivery media (ThermoFisher Scientific) supplemented with 100 ng/ml mouse SCF, 100 ng/ml human TPO and 100 ng/ml mouse Flt3 ligand, and cultured with 0.5 mM each of Accell SMART Pool of the following four double-stranded siRNA for mouse Hoxa9: CGCUUGACACUCACACUUU, UGACUAUGCUUGUGGUUCU, CUUGCAGCUUCCAGUCCAA, UGGGCAACUACUAUGUGGA, or the negative control: Accell Non-targeting Pool (ThermoFisher Scientific) for 72 hrs. Efficiency of the co-transfection was monitored by using green fluorescent dye-labeled siRNA (Accell Green Non-targeting siRNA; ThermoFisher Scientific) as an indicator. Cells were then subjected to further analysis.

### Immunoprecipitation and immunoblot analysis

Cell extracts were prepared by resuspending cell pellets in RIPA buffer consisting of 10% glycerol, 0.5% Triton X-100, 20 mM Hepes (pH 8.0), 150 mM NaCl, 1 mM EDTA, 1.5 mM MgCl_2_, and a protease inhibitor cocktail, Complete Mini (Roche Diagnostics Gmbh, Mannheim, Germany), sonicated for 30 sec on ice and centrifuged for 15 min at 15,000× g. The supernatant of the lysate was subjected to immunoprecipitation experiments with GammaBind G Sepharose (GE Healthcare, Milwaukee, WI). Proteins were separated by SDS-PAGE, transferred to Immobilon-P (Merck, Billerica, MA), immunoblotted with primary antibodies, and visualized with horseradish peroxidase-conjugated anti-rabbit or anti-mouse IgG and SuperSignal West Femto Maximum Sensitivity Substrate (ThermoFisher Scientific). The detected bands were scanned with the Image J program (NIH) and the intensity was statistically analyzed. To detect ubiquitinated molecules *in vivo*, cells were treated with MG132 (20 µM) (Peptide Institute, Osaka, Japan) for 6 hrs. For the pulse-chase labeling experiment, Geminin was labeled with [^35^S]methionine, immunoprecipitated and detected by means of autoradiography. The half-life was estimated with the least-squares method [37].

### Retrovirus-mediated gene transduction

Either the murine stem cell virus (MSCV) vector with the enhanced yellow fluorescent protein (EYFP) gene as a selection marker (MEP) or MSCV with the resistance gene for puromycin (MPI) was co-transfected with gag, pol and vesicular stomatitis virus glycoprotein (VSV-G) envelope expression plasmids into HEK-293 cells with Lipofectamine 2000 (Life Technologies). The ecotropic packaging cell line, PlatE (kindly provided by Dr. Toshio Kitamura, The Institute of Medical Science, The University of Tokyo, Tokyo, Japan) [38], was infected three to ten times with a virus, and the supernatants were concentrated by centrifugation at 6,000× g for 16 hrs to produce a high-titer helper-free retrovirus. BM were cultured for 24 hrs in DMEM supplemented with 15% FBS, 100 ng/ml mouse SCF, 100 ng/ml human TPO and 100 ng/ml mouse Flt3 ligand. The cells were then cultured with retrovirus in retronectin-coated dishes (Takara Bio, Otsu, Japan) for 72 hrs in the same medium with the addition of 5 µg/ml protamine sulfate (Sigma-Aldrich) [21],[23]. Then, the retrovirally transduced cells were subjected to further analysis.

### Analysis of cell cycle, apoptosis, Geminin protein expression and hematopoiesis

Cells were pulse-labeled with BrdU at 10 µg/ml for 45 min, permeabilized and stained by using the APC BrdU Flow Kit (BD Pharmingen, San Jose, CA). Geminin protein expression in each phase of the cell cycle was detected by additional immuno-staining with a rabbit polyclonal antibody raised against glutathione S transferase (GST)-Geminin [21]. Cell sorting analysis was performed on the FACSCalibur flow cytometer and FACSAria II cell sorter (BD Biosciences Immunocytometry Systems, San Jose, CA). Cell sorting conditions were pre-determined by using wild-type BM preceding to each of the examinations. Flow cytometry data were analyzed by CellQuest (BD Bioscience Immunocytometry Systems) or FlowJo (Tree Star, Ashland, OR). The hematopoietic capability of BM was assessed by using the clonogenic and repopulating activities. Clonogenic activity was assayed as follows: BM were cultured in DMEM supplemented with 15% FBS, 100 ng/ml mouse SCF, 100 ng/ml human TPO and 100 ng/ml mouse Flt3 ligand for 24 hrs. The cells were then infected with retroviruses. After washing in MEM alpha medium (Life Technologies), 1×10^4^ cells were resuspended in methylcellulose semisolid medium (Methocult M3231; Stem Cell Technologies, Vancouver, Canada), and plated in 35-mm culture dishes in the presence of 10 ng/ml mouse SCF, 10 ng/ml mouse GM-CSF, 10 ng/ml mouse IL-3 (R&D systems) and 3 U/ml human EPO (Chugai Pharmaceutical, Tokyo, Japan). Colonies were counted after 7 days of culture under an inverted microscope. All of the cells were harvested from methylcellulose medium, washed with 1×PBS, and then replated in the same medium (1×10^4^ cells/35-mm dish). Colony scoring and replating were repeated every 7 days. Three-month and 1-month repopulating activities were assayed by injecting 1×10^6^ EYFP^+^ retrovirally transduced BM and 2×10^5^ competitors into C57Bl/6 congenic mice lethally irradiated with 9.0 Gy administered in a single dose from a ^60^Co γ-ray source. EYFP^+^-multi-lineage cells were examined 1 and 3 months after the transplantation [21],[23],[39].

### Reconstitution of RDCOX in *Spodoptera frugiperda* cells (Sf9) and *in vitro* ubiquitination assay

Sf9 (kindly provided by Akira Kikuchi, Osaka University Graduate School of Medicine, Suita, Japan) [40] were cultured in Grace's insect cell culture medium (Life Technologies) supplemented with 10% FBS and 0.06% tryptose phosphate broth-Bacto (BD Pharmingen). His6-tagged Roc1 cDNA was inserted into pV-IKS to produce the GST-fusion product, and cDNAs for Ddb1, Cul4a, and Flag-tagged Hox derivatives were inserted into pVL1392. The vectors were co-transfected into Sf9 with a linearized BaculoGold baculovirus DNA (BD Pharmingen) for viral particle formation by means of Cellfectin (Life Technologies). Sf9 were infected with high-titer viruses, and 72 hrs post-infection the cells were washed with cold PBS and suspended in homogenizing buffer (20 mM Tris [pH 7.9], 4 mM MgCl_2_, 500 mM NaCl 0.4 mM EDTA, 2 mM DTT, 20% glycerol, 0.1% NP40, 1 mM ZnCl_2_, with Complete Mini). The suspension was homogenized and centrifuged at 15, 000× g for 10 min, after which the supernatant was subjected to Glutathione affinity column chromatography (Glutathione Sepharose 4 Fast Flow; GE Healthcare). Recombinant myc-Geminin, which was tagged with His6 and myc at the N- and C-terminal portions respectively, was produced in *Escherichia coli*, BL21, and purified by means of cobalt affinity chromatography (Co-Agarose) (Wako Pure Chemical, Osaka, Japan). For the *in vitro* ubiquitination assay, the recombinant myc-Geminin was incubated in a 20 µl reaction mixture containing 50 mM Tris-HCl (pH 7.9), 5 mM MgCl_2_, 0.6 mM DTT, 2 mM ATP, 0.1 µg ubiquitin activating enzyme E1 (Wako Pure Chemical), 0.6 µg ubiquitin conjugating enzyme UbcH5c (Enzo Life Science, Plymouth Meeting, PA), and 1 µg biotin-tagged ubiquitin (biotin-ubiquitin) or 10 µg ubiquitin (Boston Biochem, Cambridge, MA) and the purified recombinant complex. In the reaction with biotin-ubiquitin, ubiquitin was also added to the reaction at the ratio of biotin-ubiquitin∶ubiquitin, 3∶1. After incubation at 37°C for 1 hr, the reaction product was subjected to immunoblot analysis [21],[23].

### Antibodies

Primary and secondary antibodies listed in Table S1 were used in the study.

### Statistical analysis

More than three independent experiments were performed and the data were statistically analyzed. The results are shown with SEMs. Statistical significance was determined with ANOVA tests.

## Results

### Reconstitution of RDCOX complexes and the E3 ubiquitin ligase activity for Geminin

**Figure 1:**
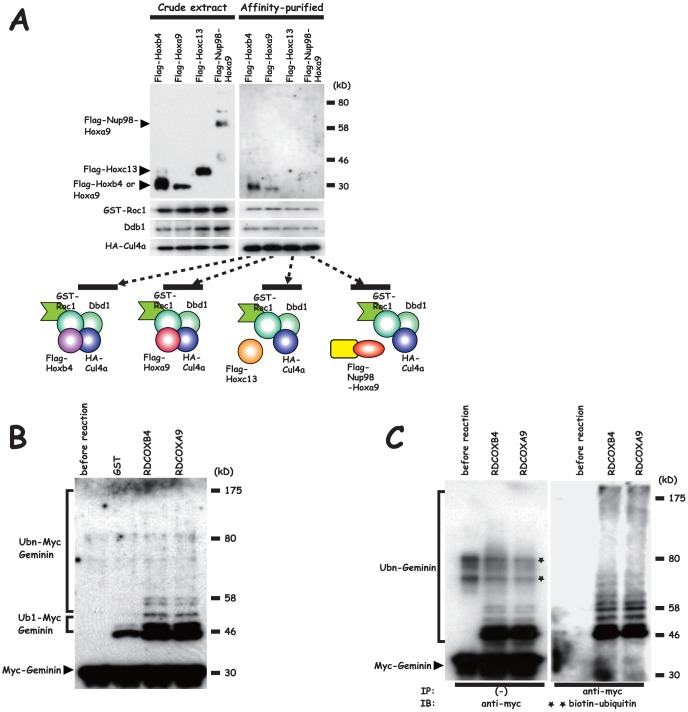
Purification of the recombinant RDCOXA9 complex from Sf9, and E3 ubiquitin ligase activity for Geminin. (**A**) Left panel: Crude extracts. Each member of the complex was detected in the crude extract by means of immunoblot analysis. Hox derivatives were detected with an anti-Flag antibody, GST-Roc1 with an anti-Roc1 antibody, Ddb1 with an anti-Ddb1 antibody and HA-Cul4a with an anti-HA antibody. Right panel: the affinity-purified. The complex with GST-Roc1 was pulled down with a glutathione affinity column chromatography. Each member was detected by means of immunoblot analysis in the pulled-down complex. Schematic representation of the complex is shown in the lower panel. The samples include GST-Roc1, Ddb1, HA-Cul4a and either of Flag-Hoxb4, Flag-Hoxa9, Flag-Hoxc13 or Flag-Nup98-Hoxa9. (**B**) E3 ubiquitin ligase activity for Geminin. The affinity-purified recombinant complex was subjected to *in vitro* ubiquitination reaction (myc-Geminin+E1+E2+ubiquitin), and the reaction product was analyzed by immunoblot analysis with an anti-myc antibody. The amount of GST-Roc1 in the RDCOXA9 complex was adjusted to that of the RDCOXB4 complex (1 µg). Ub1-Geminin, mono-ubiquitinated Geminin; Ubn-Geminin, poly-ubiquitinated Geminin. The mono-ubiquitinated Geminin bands were detected in this *in vitro* assay system even in the absence of E3 ubiquitin ligase (second lane). (**C**) *In vitro* ubiquitination reaction with biotin-tagged ubiquitin. myc-Geminin was immunoprecipitated with an anti-myc antibody after the reaction, and ubiquitinated Geminin was detected through biotin-avidin interaction. *, unspecified bands. **biotin-ubiquitin was detected with avidin vectastain.

We previously provided evidence from *in vivo* as well as *in vitro* experiments that Hoxb4 forms the RDCOXB4 complex with the Roc1-Ddb1-Cul4a ubiquitin ligase core component to down-regulate Geminin through UPS [23]. In the current study, we examined whether the Hox derivatives, Hoxa9, Hoxc13 and Nup98-Hoxa9, feature E3 ubiquitin ligase activity for Geminin similar to that of Hoxb4. To this end, we reconstituted the recombinant protein complex with the Hox derivatives in Sf9. Sf9 were co-infected with baculoviruses encoding GST-Roc1, Ddb1, HA(derived from influenza hemagglutinin A)-tagged Cul4a (HA-Cul4a) and Flag-tagged Hox derivatives. Cell extracts were prepared from Sf9, and expression of each of these components was detected by means of immunoblot analysis (Fig. 1A). We then purified (GST-Roc1)-Ddb1-(HA-Cul4a)-(Flag-Hox derivatives)[recombinant RDCOX complexes] by glutathione affinity column chromatography, and immunoblot analysis was used to examine the purified complexes for the presence of each of the components (Fig. 1A), resulting in the detection of GST-Roc1, Ddb1 and HA-Cul4a. Furthermore, Flag-Hoxa9 was detected in one of the purified complexes, which is similar to Flag-Hoxb4, while the other Hox derivatives, Nup98-Hoxa9 and Hoxc13, were not, indicating that recombinant Hoxa9 formed an RDCOX complex in Sf9 but that the other Hox derivatives did not. The affinity-purified recombinant RDCOXA9 was then subjected to an *in vitro* ubiquitination assay with purified bacterially produced recombinant myc-Geminin as a substrate [21],[23] and the reaction product was examined by means of immunoblot analysis with an anti-myc monoclonal antibody (Fig. 1B). Mobility-shifted Geminin bands were detected in the reaction products with the RDCOXA9 complex in the form of an E3 ubiquitin ligase similar to that of the reaction products with the RDCOXB4 complex. Next, an *in vitro* ubiquitination assay with biotin-ubiquitin was performed to determine whether the shifted bands were associated with ubiquitinated Geminin. Immunoprecipitation of myc-Geminin with an anti-myc polyclonal antibody after the reaction resulted in the detection of the mobility-shifted bands in the immunoprecipitate through the biotin-avidin interaction. These bands were quite similar to those detected by means of immunoblot analysis with an anti-myc monoclonal antibody (Fig. 1C), confirming that the mobility-shifted bands represented ubiquitinated Geminin. The shifted bands with high mobility, however, tended to be more sensitively detected in the immunoprecipitate as a result of the biotin-avidin interaction, probably due to polymerization of biotin-ubiquitin molecules. The detected mobility-shifted bands were quite similar to those detected in the reaction products with RDCOXB4, as previously reported by us [23], where the lower two shifted bands corresponded to mono-ubiquitinated Geminin and the other shifted bands corresponded to poly-ubiquitinated Geminin. These *in vitro* findings clearly showed that recombinant Hoxa9 forms a RDCOXA9 complex that acts as E3 ubiquitin ligase for Geminin.

### Effect of Hoxa9 on Geminin protein in HEK-293 cells

**Figure 2:**
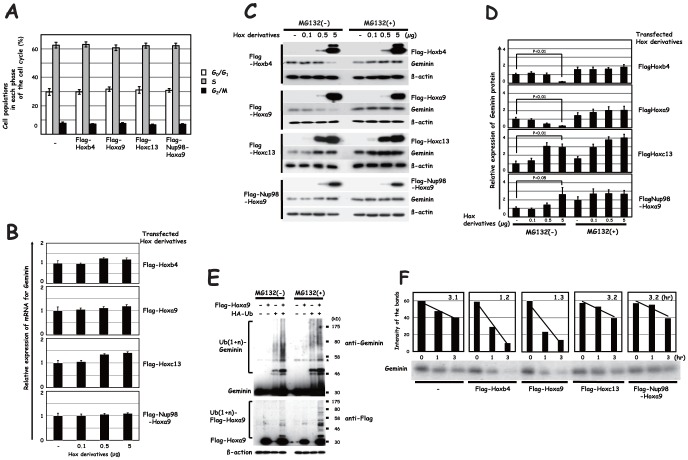
Effects of Hoxa9, Hoxc13 and Nup98-Hoxa9 transfection in HEK-293 cells. Either Flag-Hoxb4, Flag-Hoxa9, Flag-Hoxc13 or Flag-Nup98-Hoxa9 was transfected. Effect of Flag-Hoxb4 transfection was used as positive control. (**A**) Effect on the cell cycle. The percentages of cell subpopulations in each phase of the cell cycle are shown. (**B**) Effect on expression of mRNA for Geminin. mRNA for Geminin was detected by real-time PCR analysis. The amount of transfected Hox derivatives are shown in the x-axis. (**C**) Protein expression of transfected Hox derivatives and the effect on Geminin protein expression examined by immunoblot analysis. Flag-Hoxa9- or Flag-Hoxb4-induced down-regulation of Geminin was suppressed by treatment with MG132. (**D**) Quantitative analysis of Geminin protein expression in HEK-293 cells transfected with either Flag-Hoxb4, Flag-Hoxa9, Flag-Hoxc13 or Flag-Nup98-Hoxa9. Bands detected by immunoblot analysis of the three independent experiments were scanned with the Image J program (NIH). Relative expression of Geminin protein to that of ß-actin was subjected to statistical analysis. Representative findings of the immunoblot analysis were shown in Fig. 2C. (**E**) Ubiquitination of Geminin and Flag-Hoxa9. Geminin, and either Flag-Hoxa9 or HA-Ub, or both, were co-transfected. The cells were then treated with or without MG132, and were subjected to immunoblot analysis with anti-Geminin (upper panel) and anti-Flag antibodies (lower panel). (**F**) Effects of Flag-Hoxb4, Flag-Hoxa9, Flag-Hoxc13 and Flag-Nup98-Hoxa9 transfection on the stability of Geminin. [^35^S]-labeled Geminin was traced by means of autoradiography after immunoprecipitation (lower panel). The detected bands were scanned with the Image J program (NIH), and the half-life of Geminin protein was calculated (insets in the upper panel). -, an empty control vector.

**Figure 3:**
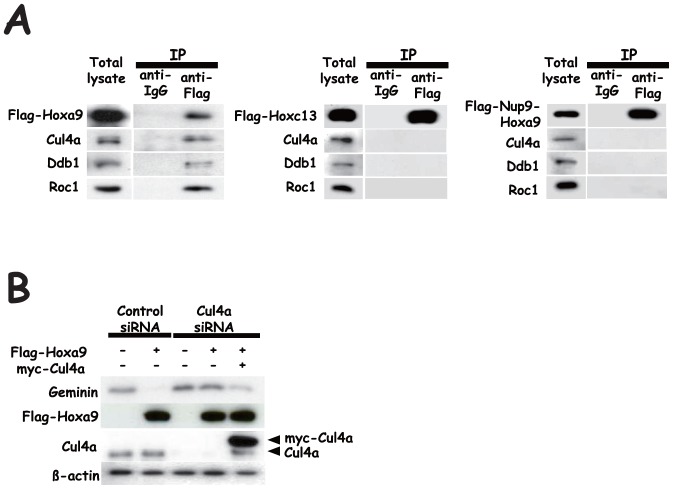
Immunoprecipitation analysis of Hox derivatives and effect of Cul4a knock-down on Hoxa9-mediated down-regulation of Geminin protein in HEK-293 cells. (**A**) Either of Flag-Hoxa9, Flag-Hoxc13 or Flag-Nup98-Hoxa9 was transfected in HEK-293 cells, and the complex formation with endogenous Cul4a, Ddb1 and Roc1 was examined by means of immunoprecipitation analysis using an anti-Flag antibody. (**B**) Cul4a siRNA was transfected, and the effect on Hoxa9-mediated down-regulation of Geminin protein was examined. Down-regulation of Cul4a by siRNA was confirmed by immunoblot analysis, and the level was restored by transfection of myc-tagged Cul4a. Endogenous Cul4a was also detected in myc-tagged Cul4a-transfected cells even if cells were pre-treated with siRNA for Cul4a probably because exogenously overexpressed mRNA for Cul4a prevented siRNA from affecting endogenous Cul4a.

We next examined the effects of the Hox derivatives Hoxb4, Hoxa9, Hoxc13 and Nup98-Hoxa9 on endogenous Geminin expression in HEK-293 cells. These derivatives were transiently transfected into HEK-293 cells and their effects on the cell cycle and Geminin expression was examined 24 hrs after the transfection by means of cell sorting, real-time PCR and immunoblot analyses. None of the Flag-Hoxb4, Flag-Hoxa9, Flag-Hoxc13 or Flag-Nup98-Hoxa9 transfections exerted any significant effect on the cell cycle (Fig. 2A), while all of them produced a slight increase in mRNA for Geminin (Fig. 2B). Interestingly, transfection of Flag-Hoxb4 or Flag-Hoxa9 reduced Geminin in a dose-dependent manner at the protein level (Fig. 2C, D). Flag-Hoxa9 transfection appeared to down-regulate Geminin protein more effectively than did Flag-Hoxb4 transfection, similar to what was observed in BM as detailed below, although we could not detect any significant difference in E3 ubiquitin ligase activity for Geminin *in vitro* nor in Geminin stability in the transfectants as described below. Down-regulation of Geminin was, on the other hand, not observed in HEK-293 cells transfected with either Flag-Hoxc13 or Flag-Nup98-Hoxa9. The down-regulation of Geminin observed in Flag-Hoxa9 transfectants was completely suppressed by treatment with MG132, an inhibitor of proteasome (Fig. 2C, D), suggesting that down-regulation of Geminin protein by Hoxa9 is mediated by UPS. Although mobility-shifted Geminin bands were detectable in HEK-293 cells co-transfected with Geminin and HA-tagged ubiquitin (HA-Ub), they appeared to be increased by the additional transfection of Hoxa9 (Fig. 2E), while treatment with MG132 produced a further increase in the mobility-shifted bands with higher molecular weights. In addition to the *in vitro* biochemical findings described above, these findings also suggest that mobility-shifted bands correspond to ubiquitinated Geminin and that the ubiquitinated Geminin with longer ubiquitin chains is preferentially degraded by proteasome. These findings suggest that poly-ubiquitination of Geminin induced by Hoxa9 down-regulates the protein level. Flag-Hoxa9, by itself, was also ubiquitinated (Fig. 2E), which is compatible with a previous report that Hoxa9 is under the regulation of UPS [41]. It, however, remains obscure whether the ubiquitinated Hoxa9 corresponds to an intermediate molecule in the reaction of the RDCOXA9 complex or Hoxa9 by itself is regulated through UPS. Since the *in vitro* findings mentioned above indicate that Hoxa9 forms the RDCOXA9 complex in conjunction with the activity of the E3 ubiquitin ligase for Geminin in Sf9, we further examined whether transfected Hoxa9 gives rise to the RDCOXA9 complex in HEK-293 cells. Transfected Flag-Hoxa9, Flag-Hoxc13 and Flag-Nup98-Hoxa9 were immunoprecipitated by means of an anti-Flag antibody, and the immunoprecipitant was subjected to immunoblot analysis with anti-Cul4a, anti-Ddb1 and anti-Roc1 antibodies. Each of these components was detected in the Flag-Hoxa9 transfectant but was not in the others (Fig. 3A), indicating that transfected Flag-Hoxa9 does indeed generate the RDCOXA9 complex with endogenous Roc1, Ddb1 and Cul4a in HEK-293 cells. For further support of the hypothesis that the Hoxa9-induced down-regulation of Geminin is mediated by the Roc1-Ddb1-Cul4a core component, we examined the effect of Flag-Hoxa9 on Geminin in Cul4a-knocked-down HEK-293 cells. As shown in Fig. 3B, siRNA–mediated knock-down of Cul4a eliminated down-regulation of Geminin induced by Flag-Hoxa9 transfection. We also found that expression of an isoform for Cul4a, Cul4b, was not affected by the treatment with siRNA for Cul4a (data not shown). To further confirm that the elimination was mediated by specific knock-down of Cul4a with siRNA, we complemented knocked-down Cul4a by means of supertransfection of myc-Cul4a into the cells, which resulted in restoration of Geminin down-regulation by the transfection of Flag-Hoxa9 (Fig. 3B). We then examined effect of the Hox derivatives on the stability of Geminin protein. HEK-293 cells were pulse-chase labeled with [^35^S]methionine *in vivo*. The time-course analysis clearly showed that the labeled Geminin was destabilized in Flag-Hoxa9-tranfected cells similar to its destabilization in Flag-Hoxb4-transfected cells (Fig. 2F). The half-lives of Geminin were estimated by densitometric analysis at 1.2 hrs in Flag-Hoxb4- and 1.3 hrs in Flag-Hoxa9-transduced cells and 3.1 hrs in pcDNA-, 3.2 hrs in Flag-Hoxc13- and 3.2 hrs in Flag-Nup98-Hoxa9-tranfected cells (Fig. 2F). Turnover of the labeled Geminin was thus accelerated by Flag-Hoxa9 transfection, which is similar to the acceleration by Flag-Hoxb4 transfection. These findings, including *in vitro* evidence obtained with the recombinant complexes described above, show good compatibility with the hypothesis that transfected Hoxa9 creates the RDCOXA9 complex and down-regulates Geminin protein through UPS.

### Effect of the transduction of Hox derivatives on Geminin expression in BM

**Figure 4:**
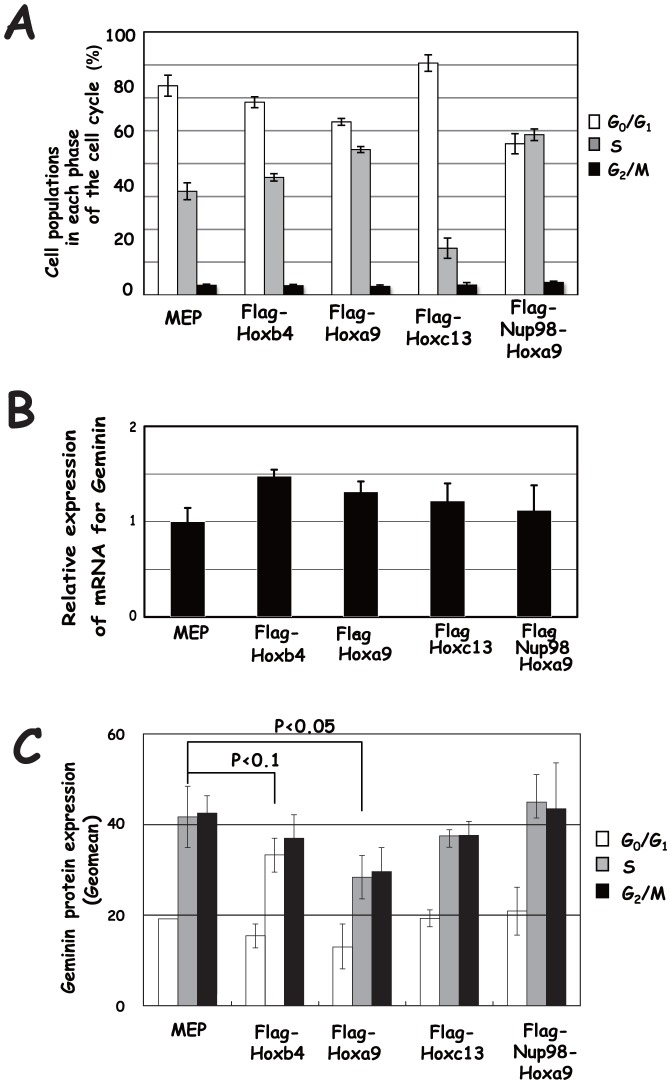
Effects of Hoxb4, Hoxa9, Hoxc13 and Nup98-Hoxa9 transduction on BM. Retrovirally transduced cells were subjected to flow cytometry analysis as well as to real-time PCR analysis. (**A**) Cell populations in each phase of the cell cycle were analyzed by cell sorting analysis. (**B**) Relative expression of mRNA for Geminin. (**C**) Geminin protein expression in each phase of the cell cycle. Geomean of the fluorescence intensity was shown. MEP, an empty control vector.

**Figure 5:**
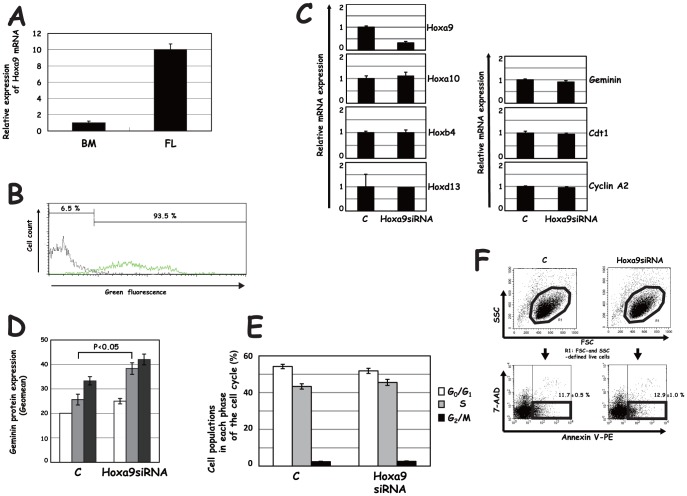
Expression of Hoxa9 in hematopoietic cells, and effect of siRNA-mediated Hoxa9 knock-down on Geminin expression in FL. (**A**) Expression of Hoxa9 mRNA in BM and FL. (**B**) Efficiency of siRNA transfection into FL. Hoxa9siRNA was co-transfected with either non-labeled non-targeting (dotted line) or green fluorescent dye-labeled non-targeting siRNA (green line) as an indicator to monitor the transfection efficiency. High transfection efficiency was confirmed by detecting expression of the indicator by flow cytometry. Rate of a green-fluorescence^+^ or green-fluorescence^−^ subpopulation (%) in the transfectants is shown in the upper part of the panel. (**C**) Effect of Hoxa9 siRNA transfection on expression of mRNA for Hoxa9, Hoxa10, Hoxb4, Hoxd13, Geminin, Cdt1 and Cyclin A2. Specific knock-down of Hoxa9 mRNA expression was confirmed by real-time PCR analysis. Hoxa9siRNA, siRNA for Hoxa9; C, non-targeting control siRNA. (**D**) Effect of siRNA transfection on Geminin protein expression in each phase of the cell cycle. Geomean of fluorescence intensity for Geminin protein was shown. (**E**) Effect of siRNA transfection on cell cycle. (**F**) Effect of siRNA transfection on apoptosis. The data from three independent experiments were subjected to the statistical analysis. The representative cell sorting data are shown.

We then examined whether the transduction of Hox derivatives exerted a similar effect on Geminin in BM. We transduced the Hox derivatives by using the MSCV vector, MEP, and examined the effect on Geminin expression [21]. Cell subpopulations in each phase of the cell cycle were fractionated by bromodeoxyuridine (BrdU) and 7-AAD, and Geminin protein expression levels were determined by means of flow cytometry (Fig. S1). Flag-Hoxb4, Flag-Hoxa9 and Flag-Nup98-Hoxa9 increased the S-phase subpopulation, while Flag-Hoxc13 reduced the S-phase subpopulation but increased the G_0_/G_1_ subpopulation (Fig. 4A). Although transduction of each of the Hox derivatives slightly increased mRNA for Geminin (Fig. 4B), protein expression levels of Geminin were reduced by the transduction of Flag-Hoxa9 in each phase of the cell cycle (p<0.05), which was similar to that of Flag-Hoxb4 (P<0.1)(Fig. 4C). Down-regulation of Geminin tended to be more effectively induced by transduction of Flag-Hoxa9 than by that of Flag-Hoxb4. The oscillation pattern of Geminin expression in the cell cycle, mainly governed by APC/C [34], was not affected. The level of Geminin expression was, on the other hand, not significantly affected by Flag-Hoxc13 or Flag-Nup98-Hoxa9 throughout the cell cycle. We then examined effect of Hoxa9 knock-down on Geminin expression. Since much higher expression of mRNA for Hoxa9 was detected in FL than in BM (Fig. 5A), siRNA for Hoxa9 (Hoxa9siRNA) was co-transfected into FL with green fluorescent dye-labeled non-targeting siRNA as an indicator. Cells were confirmed to be efficiently co-transfected by detecting expression of the indicator (93.5%)(Fig. 5B), and effect of transfection of Hoxa9siRNA on Hoxa9, Hoxa10, Hoxb4 and Hoxd13 expression and further that on Geminin, Cdt1 and Cyclin A2 expression were examined. Transfection of Hoxa9siRNA gave rise to specific down-regulation of mRNA for Hoxa9 (Fig. 5C) and up-regulation of Geminin protein in every cell-cycle phase (Fig. 5D) without significantly affecting the expression level of Geminin mRNA (Fig. 5C). We further confirmed that transfection of Hoxa9siRNA did not affect the cell cycle (Fig. 5E) and apoptosis (Fig. 5F).

### Involvement for Geminin down-regulation in the effect of Hoxa9 transduction on clonogenic activity

**Figure 6:**
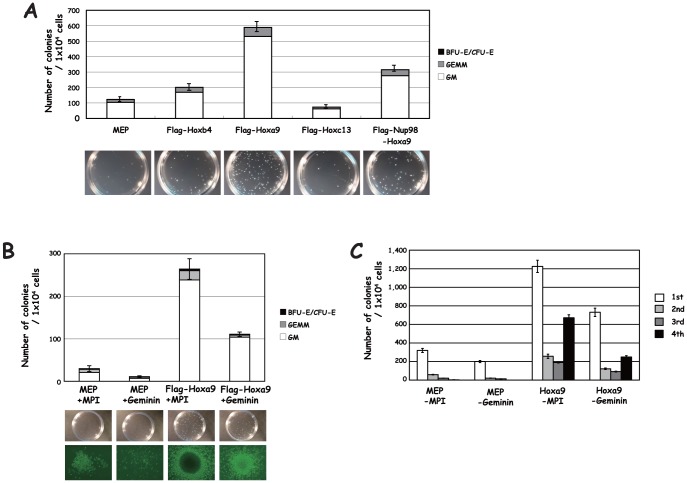
Effect on clonogenic activity. (**A**) Effect of transduction of Hox derivatives on clonogenic activity. Numbers and types of colonies are shown in the upper panel, and close-up photographs of representative colonies in the lower panel. (**B**) Effect of Geminin supertransduction on Hoxa9 transduction-mediated induction of clonogenic activity. Numbers and types of colonies are shown in the upper panel. Close-up photographs of representative colonies and images obtained with an inverted microscopy are shown in the lower panel. A cell cluster with more than 20 cells was counted as a colony under microscopy. (**C**) Effect of Geminin supertransduction on Hoxa9 transduction-mediated induction of replating activity. MEP and MPI, empty control vectors.

We next examined effect of Hox derivatives on clonogenic activity in BM (Fig. 6A). Flag-Hoxa9 transduction more strongly increased the number and size of colonies than Flag-Hoxb4 transduction and also increased the relative proportion of GM colonies. Transduction of Flag-Nup98-Hoxa9 also induced clonogenic activities but less efficiently than Flag-Hoxa9. Flag-Hoxc13 transduction resulted in larger colonies, though the frequency of colonies did not change. To examine whether down-regulated Geminin is involved in the molecular mechanism underlying the Hoxa9 transduction-mediated clonogenic induction, we supertransduced Geminin into Flag-Hoxa9-transduced BM: BM were first transduced with MEP-Flag-Hoxa9, and then supertransduced with Geminin which was cloned into the MSCV vector, MPI [23]. The supertransduced cells were then subjected to clonogenic analysis (Fig. 6B). Transduction of Geminin caused the Geminin expression level in Flag-Hoxa9-transduced cells, which was reduced throughout the cell cycle as stated earlier, to revert to a level close to that in MEP-transduced cells (data not shown). The level of restored Geminin by Geminin supertransduction was similar to that for BM from the reconstituted mice as described below. The clonogenic assay clearly showed that Geminin supertransduction efficiently reduced the clonogenic activity enhanced by Hoxa9 transduction (Fig. 6B). The number of colonies was reduced as a result of Geminin transduction, and their size was also somewhat reduced in comparison with that of Flag-Hoxa9-transduced BM. Geminin supertransduction, however, did not alter the relative frequency of the colony type compared to that in Hoxa9-transduced BM. We further examined the replating activities. Enhanced replating activity by Hoxa9 transduction was partially reduced by Geminin supertransduction (Fig. 6C).

### Effect of Geminin transduction on hematopoietic induction elicited by Hoxa9 transduction

**Figure 7:**
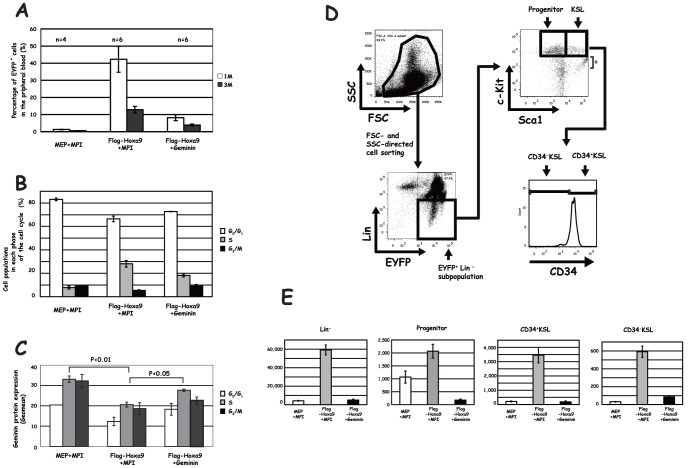
Effect of Geminin supertransduction on Hoxa9 transduction-mediated hematopoietic induction in reconstituted BM. (**A**) Repopulating activities of retrovirally transduced BM. Repopulating activities in the reconstituted mice were examined 1 month (1M) and 3 months (3M) after the injection. Since the repopulating activity was low in MEP+MPI-transduced BM, mice injected with 5-fold higher numbers of retrovirally transduced cells were used in subsequent analyses as controls. The number of recipient mice is indicated above each bar. (**B**) Cell populations in each phase of the cell cycle were analyzed by cell sorting analysis. (**C**) Geminin protein expression in each phase of the cell cycle. (**D**) Cell sorting procedure for analyzing the primitive hematopoietic cells. BM were subjected to cell sorting analysis. Primitive hematopoietic cells in EYFP^+^ cells were examined by the immunophenotype analysis. *, cells with a non-specific Sca1 signal due to the spectrum overlap (**E**) Cell numbers of Lin^−^, progenitor, CD34^+^KSL and CD34^−^KSL subpopulations in BM from the reconstituted mice. The data from three independent mice were subjected to the statistical analysis. MEP and MPI, empty control vectors.

Since Hoxa9 was shown to enhance HSC and progenitor cell activity [5], we investigated effect of Hoxa9 transduction-mediated down-regulation of Geminin on the reconstitution ability of BM. MEP-Flag-Hoxa9 was transduced into BM, and transduced cells were then injected into lethally irradiated congenic mice. One and 3 months post-injection, the peripheral blood was examined for EYFP^+^ cells. As shown in Fig. 7A, the percentage of EYFP^+^ cells in mice injected with Flag-Hoxa9-transduced BM markedly increased 1 month after the injection. Although the percentage of EYFP^+^ cells had decreased 3 months after the injection, a clear increase in the percentage of EYFP^+^ cells was detected in mice with Flag-Hoxa9-transduced BM compared to those with MEP+MPI-transduced BM. EYFP^+^ cells consisted of multi-lineage hematopoietic cell subpopulations (data not shown). Hoxa9 transduction increased the S-phase subpopulation (Fig. 7B) and decreased Geminin expression levels in each cell cycle phase (Fig. 7C). To determine the effect of returning Geminin levels to a normal range, we injected mice with Flag-Hoxa9- and Geminin-supertransduced BM in parallel. It was confirmed that the diminished Geminin expression level in each of the cell cycle phase was partially restored in BM as a result of Geminin supertransduction (Fig. 7C). The enhanced number of EYFP^+^ cells in the peripheral blood was reduced by Geminin supertransduction either 1 or 3 months after the injection (Fig. 7A). We also examined the effect of Geminin supertransduction on Hoxa9 transduction-mediated alterations in hematopoietic subpopulations by analyzing EYFP^+^ phenotypic hematopoietic stem and progenitor cell subpopulations in reconstituted mice 5 months after the injection (Fig. 7D). CD34^−^ c-Kit^+^ Sca1^+^ Lineage^−^ (CD34^−^KSL: the HSC subpopulation), CD34^+^KSL (the multipotential progenitor-cell subpopulation), c-Kit^+^ Sca1^−^ Lineage^−^ (the progenitor subpopulation) [42] or Lineage^−^(Lin^−^) subpopulations were increased by Flag-Hoxa9 transduction, which was strongly suppressed by Geminin supertransduction (Fig. 7E). These findings suggest that Geminin down-regulation induced by Hoxa9 transduction plays an important part in Hoxa9 transduction-mediated induction of HSCs and hematopoietic progenitors.

## Discussion

In this study we demonstrated that transduction of Hoxa9 down-regulated expression of Geminin protein through UPS, in both *in vitro* and *in vivo* assays. Similar to Hoxb4, Hoxa9 was observed to generate a RDCOX complex that displayed E3 ubiquitin ligase activity for Geminin *in vitro*, while Hoxc13 and Nup98-Hoxa9 did not. The stability of the Hoxa9 protein by itself, on the other hand, was previously reported to be regulated by UPS [41]. Since in this study we demonstrated that Hoxa9 formed the RDCOXA9 complex and was ubiquitinated in HEK-293 cells co-transfected with Hoxa9 and ubiquitin and we also previously reported that each member of the RDCOXB4 complex including Hoxb4 was poly-ubiquitinated in the *in vitro* ubiquitination system [23], Hoxa9 may be ubiquitinated in the RDCOXA9 complex in the course of the ubiquitination reaction to Geminin, which might also play a role in the regulation of the protein stability of Hoxa9 by itself. Nup98-Hoxa9, unlike Hoxa9, however, is reportedly not regulated by UPS [43], and this finding agrees well with ours that the complex formation of Nup98-Hoxa9 with the Roc1-Ddb1-Cul4a core complex was not detectable. Although it has been reported that Nup98-Hoxa9 up-regulates Hoxa9 expression in human CD34^+^ cord blood cells [43], we could detect neither enhanced expression of Hoxa9 in Nup98-Hoxa9-transduced BM by means of immunoblot analysis (data not shown) nor down-regulation of Geminin, which suggests that Hoxa9-mediated Geminin down-regulation is not involved in Nup98-Hoxa9-mediated hematopoietic induction and leukemogenesis.

As we reported previously, Geminin expression is high in HSCs but is down-regulated in hematopoietic progenitors with higher proliferation potential [21]. Down-regulation of Geminin may thus be important for a molecular mechanism of how transduced Hoxa9 enhanced activities of HSCs and hematopoietic progenitors. This can be accounted for by the findings that Hoxa9 transduction-mediated enhancement of repopulating, replating and clonogenic activities was suppressed by supertransduction of Geminin and that siRNA-mediated knock-down of Geminin promoted hematopoietic progenitor activity and constitutive overexpression of Geminin eliminated HSC activity as reported previously by us [21],[23]. Although we demonstrated that Hoxa9 plays a similar molecular role to that of Hoxb4, deficiency in Hoxb4 reportedly exerts a subtle effect on hematopoiesis [44], while that in Hoxa9 impairs the proliferation and repopulating ability of HSCs [45],[46]. Why was there an apparent difference in hematopoietic phenotype between mice deficient in Hoxb4 and those in Hoxa9? First, Hoxa9 is one of the most highly expressed Hox genes in primitive hematopoietic cells [47], and second, the transfection experiment involving HEK-293 cells suggested that Hoxa9 transfection tended to down-regulate Geminin more efficiently than Hoxb4, and Hoxa9 transduction more effectively down-regulated Geminin in BM. These might be the reasons why the stronger hematopoietic phenotypes appeared in Hoxa9-deficient mice and why deficiency of Hoxa cluster genes impaired hematopoiesis [47],[48] while that of Hoxb cluster genes did not [49]. On the other hand, since enhanced replating ability was only partially suppressed by Geminin supertransduction in Hoxa9-transduced BM but was almost completely in Hoxb4-transduced ones [23], there might also be difference in the down-stream target genes for these Hox genes.

Down-regulated Geminin might also give rise to genome instability through the induction of rereplication in Hoxa9-transduced cells. This induced genome instability as well as the accelerated cellular proliferation might provide a cellular background for leukemic transformation. It reportedly takes 3 to 10 months for Hoxa9-transduced BM to give rise to leukemia after the transplantation [5], which appears to be compatible with the hypothesis that Hoxa9 transduction induces genomic instability to trigger the second hits as well as provides proliferation potential. On the other hand, neither Nup98-Hoxa9 nor Hoxc13 formed a complex with Roc1-Ddb1-Cul4a according to the assay system in our study. Nup98-Hoxa9 was shown to induce transcription through direct interaction with p300/CBP [50]. Nup98-Hoxc13 is known to be involved in leukemogenesis, but transduction of Hoxc13 by itself neither formed the RDCOX complex displaying the E3 ubiquitin ligase for Geminin nor increased number of cells with clonogenic activity. Hoxc13 was previously shown to directly interact with Pu.1 to negatively regulate differentiation in murine erythroleukemia cells [51], and also to be a member of replication complexes [52]. Our findings suggest that Hoxa9 plays a role similar to that of Hoxb4 but not to that of other Hox gene derivatives, Hoxc13 and Nup98-Hoxa9, in the molecular mechanism for enhancing hematopoiesis. In addition to the transcriptional regulatory role the novel molecular function for Hoxa9 proposed in the current study may thus provide an important clue for deepening understanding of the role of Hoxa9 in hematopoiesis.

## Supporting Information

Figure S1 **Cell sorting procedure for determining Geminin protein expression level in each phase of the cell cycle.** Retrovirally transduced BM were subjected to the cell cycle analysis. Geminin expression levels (Geomean) were examined in each phase of the cell cycle.
(JPG)

Table S1 **Antibodies used in the study.**
(DOCX)
