# Supplementary figures and images for: Hoxa9 Transduction Induces Hematopoietic Stem and Progenitor Cell Activity through Direct Down-Regulation of Geminin Protein
Source: PLoS One. 2013 Jan 11;8(1):e53161. doi: 10.1371/journal.pone.0053161 (PMC3543444; doi:10.1371/journal.pone.0053161)

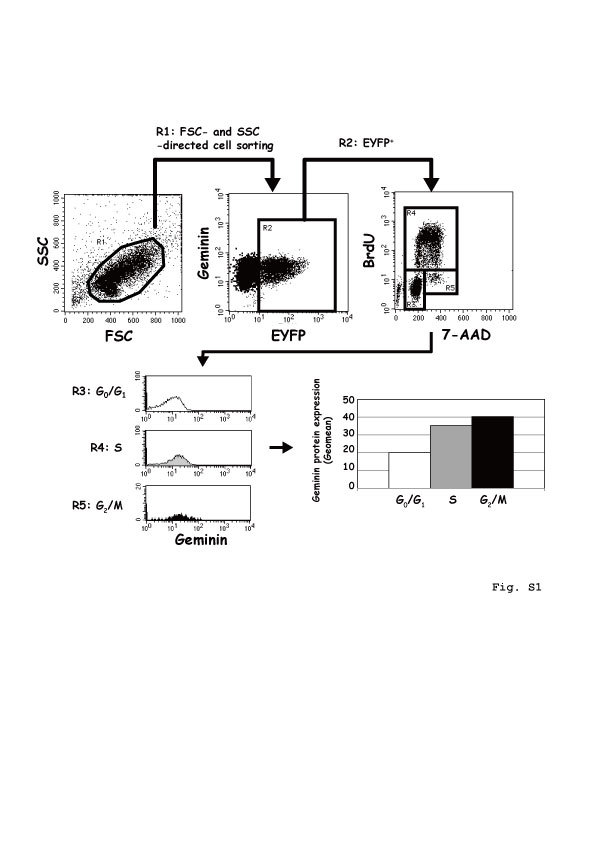

Supplement: Figure S1 — Cell sorting procedure for determining Geminin protein expression level in each phase of the cell cycle. Retrovirally transduced BM were subjected to the cell cycle analysis. Geminin expression levels (Geomean) were examined in each phase of the cell cycle. (JPG) [file pone.0053161.s001.jpg]
