# Supplementary material for: Hoxa9 Transduction Induces Hematopoietic Stem and Progenitor Cell Activity through Direct Down-Regulation of Geminin Protein
Source: PLoS One. 2013 Jan 11;8(1):e53161. doi: 10.1371/journal.pone.0053161 (PMC3543444; doi:10.1371/journal.pone.0053161)
Supplement: Table S1 — Antibodies used in the study. (DOCX) [file pone.0053161.s002.docx]

**Table S1. Antibodies used in the study.**

**Antibody** (Clone or Product number) **Species(*) Manufacturer**

**Probe-conjugated antibody**

anti-CD34+APC(RAM34) rat (m) eBioscience

anti-Sca-1/Ly-6A/E+PE-Cy7(D7) rat (m) eBioscience

anti-c-Kit

/CD117+APC-eFluor780(2B8) rat (m) eBioscience

anti-B220/CD45R+Biotin(RA3-6B2)& rat (m) eBioscience

anti-CD3ε+Biotin (145-2C11)& hamster (m) eBioscience

anti-Gr-1/Ly-6G+Biotin(RB6-8C5)& rat (m) eBioscience

anti-Mac-1α/CD11b+Biotin(M1/70)& rat (m) eBioscience

anti-TER-119+Biotin(TER-119)& rat (m) eBioscience

**Primary antibody**

anti-Flag(ANTI-FLAG M2) mouse (m) Sigma

anti-myc(A14) rabbit (p) Santa Cruz

anti-myc(9E10) mouse (m) Santa Cruz

anti-HA(12CA5) mouse (m) Roche

anti-Geminin rabbit (p) #

anti-Roc1(34-2500) rabbit (p) Life Tech.

anti-Ddb1(H-300) rabbit (p) Santa Cruz

anti-Cul4a(A300-739A) rabbit (p) Bethyl

anti-Hoxa9(sc-17155) goat (p) Santa Cruz

anti-Gapdh(GAPDH-71.1) mouse (m) Sigma

anti-β-actin(AC-74) mouse (m) Sigma

**Secondary antibody****

anti-rabbit IgG+PE(611-108-122) goat (p) Rockland

anti-rabbit IgG+HRP(1858415) goat (p) Thermo

anti-mouse IgG+HRP(1858413) goat (p) Thermo

anti-goat IgG+HRP(sc-2020) donkey (P) Santa Cruz

***** Monoclonal or polyclonal antibody is indicated by m or p, respectively.

# Polyclonal antibodies raised against the GST-fusion recombinant molecules [[21](#_ENREF_21)].

** Secondary antibodies conjugated with labels.

& Lin^-^ cells were purified by using the above-listed biotin-conjugated antibodies with Streptavidin+PerCP-Cy5.5 (BD).

Life Tech., Life Technologies; Thermo, ThermoFisher Scientific;

Sigma, Sigma-Aldrich; Roche, Roche Diagnostics Gmbh
